# (Methyl­enedinitrilo)tetra­acetonitrile

**DOI:** 10.1107/S160053681000824X

**Published:** 2010-03-10

**Authors:** Xiang-Hua Song, Mei-Li Feng, Kai Wang, Yu-Feng Li, Hong-Jun Zhu

**Affiliations:** a Department of Applied Chemistry, College of Science, Nanjing University of Technology, Nanjing 210009, People’s Republic of China

## Abstract

The mol­ecular structure of the title compound, C_9_H_10_N_6_, exhibits four cyano­methyl groups around a central N—CH_2_—N unit. In the crystal structure, mol­ecules are connected *via* inter­molecular C—H⋯N hydrogen bonds, forming a three-dimensional network.

## Related literature

For bond-length data, see: Allen *et al.* (1987 ▶). For the synthetic procedure, see: W. R. Grace & Co. (1969 ▶). For the use of the title compound in the synthesis of *N*-(phosphono­meth­yl) imino­diacetic acid, see: Obeso Caceres & Urcelay del Pozo (1991 ▶).
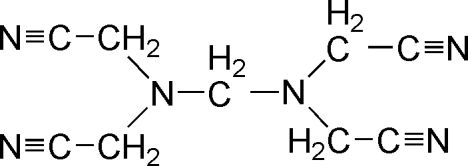


## Experimental

### 

#### Crystal data

C_9_H_10_N_6_
*M* *_r_* = 202.23
Monoclinic, 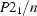

*a* = 6.743 (1) Å
*b* = 15.984 (3) Å
*c* = 10.610 (2) Å
β = 105.88 (3)°
*V* = 1099.9 (4) Å^3^
*Z* = 4
Mo *K*α radiation
μ = 0.08 mm^−1^
*T* = 293 K
0.30 × 0.20 × 0.10 mm

#### Data collection

Enraf–Nonius CAD-4 diffractometer
Absorption correction: ψ scan (North *et al.*, 1968 ▶) *T*_min_ = 0.976, *T*_max_ = 0.992
2167 measured reflections
1991 independent reflections
1396 reflections with *I* > 2σ(*I*)
*R*_int_ = 0.016
3 standard reflections every 200 reflections  intensity decay: 1%

#### Refinement

*R*[*F* ^2^ > 2σ(*F* ^2^)] = 0.047
*wR*(*F* ^2^) = 0.143
*S* = 1.00
1991 reflections
137 parameters
H-atom parameters constrained
Δρ_max_ = 0.13 e Å^−3^
Δρ_min_ = −0.14 e Å^−3^

### 

Data collection: *CAD-4 EXPRESS* (Enraf–Nonius, 1994 ▶); cell refinement: *CAD-4 EXPRESS*; data reduction: *XCAD4* (Harms & Wocadlo,1995 ▶); program(s) used to solve structure: *SHELXS97* (Sheldrick, 2008 ▶); program(s) used to refine structure: *SHELXL97* (Sheldrick, 2008 ▶); molecular graphics: *SHELXTL* (Sheldrick, 2008 ▶); software used to prepare material for publication: *SHELXTL*.

## Supplementary Material

Crystal structure: contains datablocks I, global. DOI: 10.1107/S160053681000824X/im2179sup1.cif

Structure factors: contains datablocks I. DOI: 10.1107/S160053681000824X/im2179Isup2.hkl

Additional supplementary materials:  crystallographic information; 3D view; checkCIF report

## Figures and Tables

**Table 1:** Hydrogen-bond geometry (Å, °)

*D*—H⋯*A*	*D*—H	H⋯*A*	*D*⋯*A*	*D*—H⋯*A*
C2—H2*B*⋯N4^i^	0.97	2.56	3.409 (3)	146
C4—H4*B*⋯N3^ii^	0.97	2.58	3.432 (3)	147

Symmetry codes: (i) 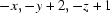; (ii) 
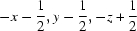
.

**Fig. 1.**
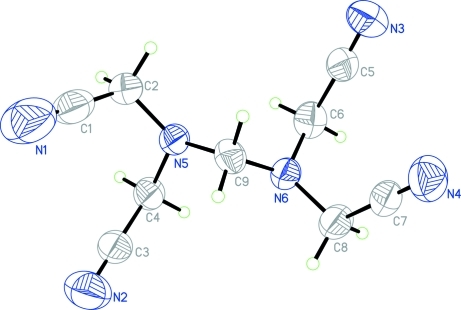
Molecular structure of the title compound showing the atom-numbering scheme. Displacement ellipsoids are drawn at the 50% probability level.

**Fig. 2.**
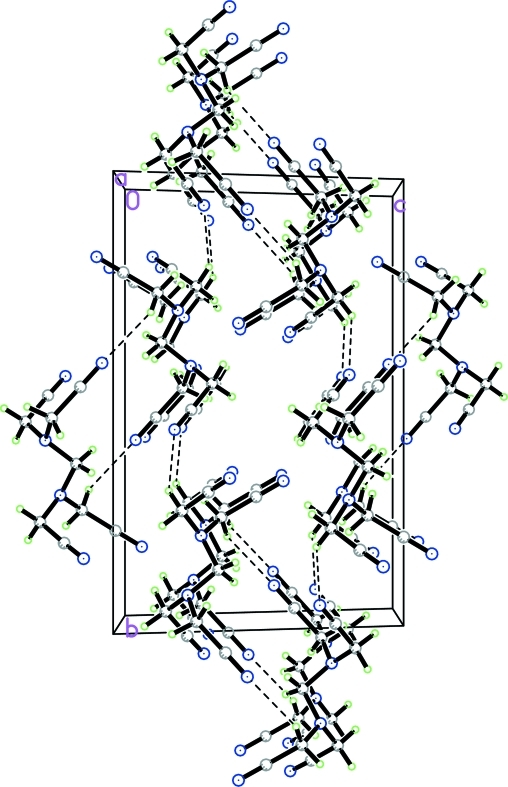
Packing diagram for (I). C—H···N hydrogen bonds are shown as dashed lines.

## supplementary crystallographic information

### Comment

The tittle compound, {[(Bis-cyanomethyl-amino)-methyl]cyanomethyl-amino}-acetonitrile is an important intermediate for the synthesis of *N*-(Phosphonomethyl) iminodiacetic acid (Obeso Caceres & Urcelay del Pozo, 1991), which can be used to synthesize glyphosphates. Herein we report the crystal structure of the title compound, (I).

The molecular structure of (I) is shown in Fig. 1, bond lengths and angles are within normal ranges (Allen *et al.*, 1987).

In the crystal of the title compound molecules are connected *via* intermolecular C—H···N hydrogen bonds to form a three dimensional network.

### Experimental

The title compound, (I) was synthesized according to a literature method (W.R. Grace &Co., 1969). Crystals were obtained by dissolving compound (I) (1.5 g) in methanol (25 ml) and evaporating the solvent slowly at room temperature for about 8 d.

### Refinement

H atoms were positioned geometrically, with C—H = 0.97 Å and constrained to ride on their parent atoms, with *U*_iso_(H) = 1.5*U*_eq_(C).

### Crystal data

**Table:**

C_9_H_10_N_6_	*F*(000) = 424
*M**_r_* = 202.23	*D*_x_ = 1.221 Mg m^−^^3^
Monoclinic, *P*2_1_/*n*	Mo *K*α radiation, λ = 0.71073 Å
Hall symbol: -P 2yn	Cell parameters from 25 reflections
*a* = 6.743 (1) Å	θ = 10–13°
*b* = 15.984 (3) Å	µ = 0.08 mm^−^^1^
*c* = 10.610 (2) Å	*T* = 293 K
β = 105.88 (3)°	Block, colorless
*V* = 1099.9 (4) Å^3^	0.30 × 0.20 × 0.10 mm
*Z* = 4	

### Data collection

**Table:**

Enraf–Nonius CAD-4 diffractometer	1396 reflections with *I* > 2σ(*I*)
Radiation source: fine-focus sealed tube	*R*_int_ = 0.016
graphite	θ_max_ = 25.3°, θ_min_ = 2.4°
ω/2θ scans	*h* = 0→8
Absorption correction: ψ scan (North *et al.*, 1968)	*k* = 0→19
*T*_min_ = 0.976, *T*_max_ = 0.992	*l* = −12→12
2167 measured reflections	3 standard reflections every 200 reflections
1991 independent reflections	intensity decay: 1%

### Refinement

**Table:**

Refinement on *F*^2^	Secondary atom site location: difference Fourier map
Least-squares matrix: full	Hydrogen site location: inferred from neighbouring sites
*R*[*F*^2^ > 2σ(*F*^2^)] = 0.047	H-atom parameters constrained
*wR*(*F*^2^) = 0.143	*w* = 1/[σ^2^(*F*_o_^2^) + (0.085*P*)^2^] where *P* = (*F*_o_^2^ + 2*F*_c_^2^)/3
*S* = 1.00	(Δ/σ)_max_ < 0.001
1991 reflections	Δρ_max_ = 0.13 e Å^−^^3^
137 parameters	Δρ_min_ = −0.14 e Å^−^^3^
0 restraints	Extinction correction: *SHELXL97* (Sheldrick, 2008), Fc^*^=kFc[1+0.001xFc^2^λ^3^/sin(2θ)]^-1/4^
Primary atom site location: structure-invariant direct methods	Extinction coefficient: 0.112 (10)

### Special details

**Table:**

Geometry. All esds (except the esd in the dihedral angle between two l.s. planes) are estimated using the full covariance matrix. The cell esds are taken into account individually in the estimation of esds in distances, angles and torsion angles; correlations between esds in cell parameters are only used when they are defined by crystal symmetry. An approximate (isotropic) treatment of cell esds is used for estimating esds involving l.s. planes.
Refinement. Refinement of *F*^2^ against ALL reflections. The weighted *R*-factor wR and goodness of fit *S* are based on *F*^2^, conventional *R*-factors *R* are based on *F*, with *F* set to zero for negative *F*^2^. The threshold expression of *F*^2^ > σ(*F*^2^) is used only for calculating *R*-factors(gt) etc. and is not relevant to the choice of reflections for refinement. *R*-factors based on *F*^2^ are statistically about twice as large as those based on *F*, and *R*- factors based on ALL data will be even larger.

### Fractional atomic coordinates and isotropic or equivalent isotropic displacement parameters (Å^2^)

**Table:**

	*x*	*y*	*z*	*U*_iso_*/*U*_eq_	
C1	−0.0963 (3)	0.70909 (15)	0.4827 (2)	0.0615 (6)	
N1	−0.0281 (3)	0.67348 (19)	0.5772 (2)	0.1033 (9)	
C2	−0.1891 (3)	0.75550 (13)	0.3610 (2)	0.0500 (5)	
H2A	−0.2811	0.7179	0.3005	0.060*	
H2B	−0.2727	0.8002	0.3813	0.060*	
N2	0.3724 (3)	0.66055 (14)	0.3957 (2)	0.0783 (7)	
C3	0.2363 (3)	0.69176 (13)	0.3233 (2)	0.0528 (6)	
N3	−0.2530 (3)	1.06022 (13)	0.3005 (2)	0.0812 (7)	
C4	0.0600 (3)	0.73457 (12)	0.23192 (19)	0.0472 (5)	
H4A	0.1101	0.7652	0.1679	0.057*	
H4B	−0.0362	0.6926	0.1851	0.057*	
N4	0.3020 (3)	1.08661 (11)	0.45391 (19)	0.0650 (6)	
N5	−0.0489 (2)	0.79180 (9)	0.29447 (15)	0.0444 (4)	
C5	−0.1757 (3)	1.01621 (13)	0.2444 (2)	0.0563 (6)	
N6	0.1060 (2)	0.91629 (9)	0.25246 (15)	0.0451 (4)	
C6	−0.0768 (3)	0.95691 (13)	0.1736 (2)	0.0560 (6)	
H6A	−0.1765	0.9142	0.1338	0.067*	
H6B	−0.0411	0.9868	0.1032	0.067*	
C7	0.2980 (3)	1.03432 (12)	0.3814 (2)	0.0480 (5)	
C8	0.2920 (3)	0.96600 (12)	0.2853 (2)	0.0555 (6)	
H8A	0.3094	0.9905	0.2053	0.067*	
H8B	0.4083	0.9292	0.3202	0.067*	
C9	0.0716 (3)	0.86329 (11)	0.35568 (18)	0.0457 (5)	
H9A	0.2025	0.8449	0.4129	0.055*	
H9B	−0.0018	0.8941	0.4076	0.055*	

### Atomic displacement parameters (Å^2^)

**Table:**

	*U*^11^	*U*^22^	*U*^33^	*U*^12^	*U*^13^	*U*^23^
C1	0.0474 (12)	0.0761 (15)	0.0674 (15)	0.0003 (11)	0.0266 (11)	0.0082 (13)
N1	0.0719 (15)	0.150 (2)	0.0938 (17)	0.0147 (15)	0.0319 (13)	0.0500 (17)
C2	0.0450 (11)	0.0465 (11)	0.0627 (12)	0.0006 (9)	0.0219 (10)	0.0000 (9)
N2	0.0670 (13)	0.0839 (15)	0.0872 (15)	0.0192 (12)	0.0263 (12)	0.0043 (12)
C3	0.0534 (13)	0.0451 (11)	0.0675 (13)	0.0022 (10)	0.0292 (11)	−0.0072 (10)
N3	0.0713 (14)	0.0637 (13)	0.1181 (18)	0.0208 (11)	0.0419 (13)	0.0157 (12)
C4	0.0527 (11)	0.0397 (10)	0.0530 (11)	−0.0015 (9)	0.0210 (10)	−0.0084 (9)
N4	0.0758 (13)	0.0522 (11)	0.0720 (12)	−0.0069 (10)	0.0287 (10)	−0.0107 (10)
N5	0.0462 (9)	0.0358 (8)	0.0559 (9)	−0.0021 (7)	0.0218 (8)	−0.0058 (7)
C5	0.0470 (12)	0.0459 (12)	0.0764 (15)	0.0039 (10)	0.0176 (11)	0.0155 (11)
N6	0.0438 (9)	0.0388 (8)	0.0552 (9)	−0.0052 (7)	0.0176 (7)	−0.0047 (7)
C6	0.0596 (13)	0.0518 (12)	0.0559 (12)	−0.0032 (10)	0.0148 (10)	0.0026 (10)
C7	0.0479 (12)	0.0408 (11)	0.0590 (12)	−0.0067 (9)	0.0210 (10)	−0.0010 (9)
C8	0.0529 (13)	0.0479 (11)	0.0733 (14)	−0.0092 (10)	0.0303 (11)	−0.0149 (10)
C9	0.0514 (11)	0.0352 (10)	0.0510 (11)	0.0026 (9)	0.0151 (9)	−0.0056 (8)

### Geometric parameters (Å, °)

**Table:**

C1—N1	1.135 (3)	N5—C9	1.449 (2)
C1—C2	1.472 (3)	C5—C6	1.478 (3)
C2—N5	1.447 (2)	N6—C6	1.441 (2)
C2—H2A	0.9700	N6—C8	1.445 (2)
C2—H2B	0.9700	N6—C9	1.452 (2)
N2—C3	1.138 (3)	C6—H6A	0.9700
C3—C4	1.480 (3)	C6—H6B	0.9700
N3—C5	1.136 (3)	C7—C8	1.487 (3)
C4—N5	1.443 (2)	C8—H8A	0.9700
C4—H4A	0.9700	C8—H8B	0.9700
C4—H4B	0.9700	C9—H9A	0.9700
N4—C7	1.132 (2)	C9—H9B	0.9700
			
N1—C1—C2	178.7 (2)	C8—N6—C9	116.50 (16)
N5—C2—C1	116.91 (16)	N6—C6—C5	115.26 (17)
N5—C2—H2A	108.1	N6—C6—H6A	108.5
C1—C2—H2A	108.1	C5—C6—H6A	108.5
N5—C2—H2B	108.1	N6—C6—H6B	108.5
C1—C2—H2B	108.1	C5—C6—H6B	108.5
H2A—C2—H2B	107.3	H6A—C6—H6B	107.5
N2—C3—C4	178.2 (2)	N4—C7—C8	179.6 (2)
N5—C4—C3	114.24 (16)	N6—C8—C7	115.34 (16)
N5—C4—H4A	108.7	N6—C8—H8A	108.4
C3—C4—H4A	108.7	C7—C8—H8A	108.4
N5—C4—H4B	108.7	N6—C8—H8B	108.4
C3—C4—H4B	108.7	C7—C8—H8B	108.4
H4A—C4—H4B	107.6	H8A—C8—H8B	107.5
C4—N5—C2	116.91 (15)	N5—C9—N6	107.91 (14)
C4—N5—C9	114.36 (14)	N5—C9—H9A	110.1
C2—N5—C9	117.30 (15)	N6—C9—H9A	110.1
N3—C5—C6	178.4 (2)	N5—C9—H9B	110.1
C6—N6—C8	116.11 (16)	N6—C9—H9B	110.1
C6—N6—C9	114.43 (15)	H9A—C9—H9B	108.4
			
N1—C1—C2—N5	160 (12)	N3—C5—C6—N6	−68 (9)
N2—C3—C4—N5	−26 (7)	C6—N6—C8—C7	71.1 (2)
C3—C4—N5—C2	−77.5 (2)	C9—N6—C8—C7	−68.2 (2)
C3—C4—N5—C9	65.1 (2)	N4—C7—C8—N6	−92 (32)
C1—C2—N5—C4	70.3 (2)	C4—N5—C9—N6	69.05 (19)
C1—C2—N5—C9	−71.2 (2)	C2—N5—C9—N6	−148.50 (16)
C8—N6—C6—C5	−77.2 (2)	C6—N6—C9—N5	68.47 (19)
C9—N6—C6—C5	63.0 (2)	C8—N6—C9—N5	−151.47 (15)

### Hydrogen-bond geometry (Å, °)

**Table:**

*D*—H···*A*	*D*—H	H···*A*	*D*···*A*	*D*—H···*A*
C2—H2B···N4^i^	0.97	2.56	3.409 (3)	146
C4—H4B···N3^ii^	0.97	2.58	3.432 (3)	147

Symmetry codes: (i) −*x*, −*y*+2, −*z*+1; (ii) −*x*−1/2, *y*−1/2, −*z*+1/2.
